# The Endemic Fever of the Eastern Shore*Read before the Richmond Academy of Medicine and Surgery, January 15, 1895.

**Published:** 1896-03

**Authors:** Geo. W. LeCato

**Affiliations:** Wachapreague, Accomac County, Va.


### THE ENDEMIC FEVER OF THE EASTERN SHORE.*

BY GEO. W. LECATO, M. D., WACHAPREAGUE, ACCOMAC CO., VA.

MR. PRESIDENT AND FELLOWS:—At the request of the chair, I have selected, as the title of this paper, a subject I trust will be none the less interesting because commonplace and familiar—"The Endemic Fever of the Eastern Shore."

Twenty-five years ago, the above title would have suggested the discussion of a disease differing materially in most of its essentials from that to which it now applies; for it is absolutely true, as we all know, that, our present endemic of continued fever has not only quite supplanted the old types of our former periodical endemic, but is doubtless increasing in prevalence year by year. This fact naturally attaches a certain practical importance to its study, and furnishes my excuse for this subject. What I may have to say, however, will be merely cursory, embracing mainly such views as have been drawn from my personal observation, leaving you to take for granted the doctrines of ætiology, pathology, and treatment commonly accpted by the profession.

In the first place, I shall state, as my individual conviction, that all forms of continued fever prevailing on the Eastern Shore are of one origin, and due to a uniform specific poison. In other words, I believe that all the forms of our present endemic may be safely summed up under the title of typhoid fever—a specific disease, varying from time to time in many of its salient features, like other morbid entities, mainly as the result of peculiar local environment, systemic condition and methods of treatment. This idea is admittedly true of most diseases. No one would expect, at the present day, that pneumonia would recover, as a rule, under the profoundly sedative treatment of Kasori or the sanguinary lancet of the phlebotomists of former days. But statistics prove that the success of our fathers, in the treatment of this disease, was quite equal to that of ours. Pneumonia must have changed type in some particular essential; and yet we

*Read before the Richmond Academy of Medicine and Surgery, January 15, 1895.

have the sub-crepitant rale, pulmonic consolidation, the rusty sputum, occasional pulmonary œdema, and even purulent infiltration—the whole sum of pathological sequence. According to my observation, we have, in the continued fever of to-day, as a rule, the prodromic malaise; the tendency to epistaxis and other hæmorrhages; marked sensitiveness of the bowel, in spite of constipation; the diurnal cycle of temperature; even rose spots in some cases; occasional perforations of the bowel—in fact, the well-known evidences of a peculiar specific poison, expending its local force in the right iliac region, and in the presence of which our anti-periodic sheet anchor is worse than a delusion. Granted that in private practice we not often are permitted the post-mortem evidences of Peyerian inflammation and ulceration; so complete a picture leaves us little room to doubt that the fever of to-day is the genuine *enteric* of Geo. B. Wood, the *dothinenteritis* of Bretonneau and the *typhoid* of Louis.

True, in an experience of thirty years, I have recognized the disease under varying manifestations. We find at the present day less coma-vigil and muttering delirium, as a rule; we have less trouble to restrain the bowel; cases do not run so rapidly to what is known as the typhoid state; in a word, there appear to be more mild attacks now than formerly. How much of this may be due to a recent modification in the intensity of the poison, and how much to the present vogue of withholding active medication, may be an open question; but my observation has never led me to believe the essential nature of the disease has sufficiently changed to entitle it to a new name.

I am sure I have never recognized a typical case of Dr. Woodward's so-called typho-malarial fever. As a matter of fact, I have not observed the two distinct types that enter into this supposed hybrid, manifesting themselves unmistakably in the same individual at the same time. I think I have never seen the one type run into, or consecutively follow the other. Finally, I have never known any case of so-called typho-malaria to be cut short, or even modified by anti-periodic treatment. Rather, on the contrary, I have been led to suspicion that these two poisons are so specifically distinct as to be in some way, mutually antagonistic. Certain it is, however explained, that on the Eastern Shore, as elsewhere, the presence of one type, as the endemic, materially qualifies the prevalence of the other. I can recall a period in my earlier practice when continued fever, in Accomac and Northampton, was a comparatively rare disease. The unfortunate reputation the peninsula had earned at that day as a hotbed for malarious poisoning has not yet been lost, though, as we all know, no longer deserved. If we accept the so-called typhoid germ as the ætiological factor in the production of continued fever, and the micro-organism of Laveran as the essential element in paludal poisoning, it would be interesting and instructive to demonstrate the correctness or absurdity of the convictions I have stated; but, so far as I know, these important demonstrations have not been made.

Of the peculiar character of the typhoid fever poison, we have, at present, only a broad field of speculation and the wonderful revelations of the microscope. Unfortunately, it may be said of the fashionable microbe theories, that while they have enlarged our learning, they have not greatly increased our practical wisdom. Does the disease really depend upon a microbe? If so, what are the peculiar conditions of its generation? Where does it mainly inhabit—the food we eat, the water we drink, or the air we breathe? And what peculiar change of local condition will explain how and why the typhoid germ of to-day should have so completely supplanted the miasmatic germ of thirty years ago? These are all practical questions of the greatest importance to us as practitioners—questions for which we are vainly seeking answers, not only from our own observation, but from the scientific investigations of others; for, locked up in these mysteries are hidden the problems of prevention and cure. Medical literature teems with plausible theories; you pay your money and you take your choice. Practically, the solution appears as remote as before. I confess that, individually, I have inclined to many different theories along this line. For instance, I had found typhoid so often among families drinking out of polluted and even filthy wells, I have become a confessed convert to the poisoned water theory. Ultimately, I had a patient who was herself a crank on this very subject. For months no single drop of water had been allowed to pass to her stomach that had not previously been boiled. Strange to say, she was the only member of her large family stricken. I can recall a family in which every member was afflicted *seriatim*, who got their water from a driven pump thirty feet under ground, the pump itself, situated on the highest elevation of the premises.

On the Eastern Shore, at least, the water supply derived from underground springs of higher or lower level, evidently has no connection with the prevalence of typhoid, as has appeared in other situations.

Another fact, hard to reconcile with the trend of our accepted theories, is that the poison is not necessarily bred in filth. The lady of the household last referred to, was an uncompromising crank on the extremes of cleanliness. Indeed, on general principles, it may be said that the higher the civilization, the more obnoxious we become to typhoid infection; certainly, the poor and squalid appear to have no peculiar claims of class distinction to the disease. And so, unfortunately, some practical negation always appears to shut us off in the line of any theory we may attempt to pursue. For the present, to be candid, I confess I am entertaining no very settled notions respecting the ætiology of typhoid fever. I am inclined to think we know as little of practical value on this subject as did our fathers. They hinted darkly, in their day, at certain poisonous "humors" manufactured in the great laboratory of nature, under laws of chemical affinity beyond their chemistry. In the later wisdom of our day, we call these results ptomaines, without being able to explain the chemical processes involved in their generation. The guess of our fathers was probably a cloak for their ignorance, that fitted as becomingly as the newer cut of a later day, and, doubtless, they wore it as honestly. And I am verily coming to believe, in my own mind, the time is not distant when ptomaine—the old "humor" of our fathers—will play a stronger role in fashionable ætiology than the present, popular, and omnipotent microbe. History repeats itself in many divisions of human research. It may be we are already drifting backwards towards the theory of our fathers, to take new bearings from old landmarks and lay out new lines of research, not so much in the domain of bacteriology, as in the expanding and limitless field of spontaneous toxicology.

I have never believed for a moment in the contagion of typhoid fever. It may be infectious in certain ways not well understood. Its specific poison is doubtless localized, and those obnoxious to it, exposed to like conditions, experience like results. I think I have never discovered stronger evidences of the contagiousness of this disease than apply with equal force to malarious fevers and even common colds.

I shall not consume a moment with reference to its symptomatology. We all agree that the diagnosis is often difficult and sometimes impossible in the earlier stages; but that the thermometer may be safely trusted to settle the doubt even short of those characteristic features common to its full development. And, in the matter of prognosis, I shall simply say there is no disease known to us of so grave a character, involving so long a course of continued pyrexia, and subject to so many complications and accidents, that so often ends in complete recovery. Still, the maxim of prognosis laid down by the older writers has probably never been improved upon, namely, that while no attack is so desperate as to encourage despair, there is none so mild as to be without danger.

What I may say of the treatment will be in nowise exhaustive—having reference mainly to my own views and practice—not because I wish to appear egotistic, but because I desire to invite discussion, to draw out a comparison of opinions and experiences for my own instruction as well as yours. I have held for many years that the professional world should, long ago, have reared a monument to that philosopher, Pitcairn, who, when asked what he thought of a certain treatise on the cure of fevers, replied, "I do not like fever cures. You may guide a fever; you cannot cure it. What would you think of a pilot who attempted to quell a storm? Either position is equally absurd. In the storm, you steer the ship as well as you can; in a fever, you can only employ patience and judicious measures to meet the difficulties of the case." Perhaps the application of this doctrine, limited to the management of typhoid fever, has not only gone a long way to make the fever of our day a milder disease than formerly, but has really involved the explanation of our present success. Until we unravel the mysteries before alluded to, typhoid will probably remain in the class of so-called self-limited diseases. However revolting to our sense of professional pride, we may as well admit there is no weapon known to our science that will strike down the malady at any period in its progress, or materially abridge its duration. Among the painful reminiscences of my earlier professional life, I recall to this day the hesitation with which I accepted this fact. And yet, what serious disease is there in which more may be done to relieve suffering, ward off danger and ultimately lead the exhausted victim to recovery?

I have an abiding faith in the thorough, but careful cleansing of the intestinal canal as a preliminary, early treatment. The bowel is to be the battle-ground, and the ileum to bear the main brunt of the contest. If this is not the avenue through which the poison originally reaches the circulation, it is certainly the outlet of depuration, and consequently, a dangerous seat of auto-infection. Here the long series of morbid processes are to be carried on, whether we incline to the modern theories of microbic multiplication or the older notions of chemical putrefaction and fermentation. But the choice of means to this end is a matter of grave importance. My own experience is that the most unpromising cases falling under our observation are those that have been doctored at home with compound cathartic pills and other irritant purgatives. One of the earliest deaths I have ever seen in the disease, followed two or three doses of croton oil, administered with the avowed purpose of "touching" an innocent and falsely accused liver. In spite of some recent journalistic assertions, I should not dare attempt the use of any purgative irritant to either the mucous or muscular coat of the bowel. I usually give one or two small doses of calomel, following with salines and large enemata to flush the bowel. I am still old-fashioned enough to believe that calomel unloads the portal circulation, that it stimulates glandular excretion, and that bile limits intestinal fermentation.

An imperative necessity is the regulation of the diet. The victim has a long and doubtful struggle to encounter; the vital forces must be provided for. And yet, states of abnormal temperature impair and even suspend the digestive powers. Solid food is rarely digested, and if it passes into the bowel, it must, necessarily, become not only an irritant, but if you please, a culture field for microbes, or a fermenting mass that only add to the tympany, diarrhœa, and consequent pyrexia. Fluids are more easily absorbed and furnish little residual bulk. The practice laid down in some of the English hospitals to give no food during the fever, that will not easily pass through the meshes of a fine sieve is, at least, safe. I encourage the free use of water, plain, medicated, and flavored. I believe in its refrigerant and depurative action.

Is there anything to be done to limit the propagation of microbes, or abate the tendency to putrefactive changes in the bowel and blood? This is a very interesting, practical question, and medical opinion is in nowise agreed upon the answer. A number of years ago, I began to give what we know as carbolized iodine, partly on the theory of its germicidal properties, mainly because of the professional policy that demands in all cases some sort of medication. I shall not tire you with any notes on this subject, but will simply say that without expecting marked results, my experience has confirmed my confidence in the remedy. Recent observation has inclined me to the belief that the phenic acid in this combination is the valuable factor, and I usually leave out the iodine now because it renders the acid less acceptable to the taste. There are few patients who object to small doses of the acid, properly diluted and flavored, and to get its results, it must be regularly and steadily continued day by day, from early morning until late bedtime. I never give medicine or food during the night, except to meet the demands of a pressing indication. While this remedy is evidently not a direct antipyretic, experience proves that after its administration for several days, the temperature falls, as a rule; the diarrhæa moderates, the tympany lessens. Recent studies of the physiological effects of phenol appear to indicate that it is not only an antiseptic, germicide and local anæsthetic, but a vaso-motor excitant; Bert and Jolyet claiming that it acts like strychnia on the excitability of the medulla. If this be true, it stands on physiological grounds as an ideal remedy in this and other low forms of fever due to septic poison. This view accords with my own experience. Dr. Robie Wood, of New York, has lately claimed that it tends to prevent the falling of the hair.

The treatment of high temperatures has been, for the past several years, one of the most interesting questions connected with the management of fever. In private practice, the Brandt method of cold baths is an awkward and bunglesome recourse which may be simply eliminated from present consideration. While I might find a case in which I should be tempted to employ it, I have not yet found such a case. I rarely use any internal medicine for the reduction of temperature. I experimented in my own individual case during an attack of this disease with some doses of the coal tar derivatives, and I shall never attempt this treatment again. My personal conviction is, that under the readings of the thermometer, we are apt, in this day, to grow unnecessarily apprehensive in the matter of temperature, and lose sight of other indications hardly less important. It is undoubtedly true that high temperatures coincide with desperate conditions, but as to which may be the cause and which the effect is always a question of practical interest. For instance, an excessive temperature induced by a mass of fermenting milk-curds in the bowel could not be scientifically and appropriately treated with a cold bath. And it is wise, in all cases of hyperpyrexia, to investigate for any possible condition that may explain the rise of temperature with the hope of being able to apply treatment to the cause rather than the effect.

In spite of the commonly accepted view of damage to the integrity of animal tissues under high temperatures, I believe our apprehensions are in danger of being overexcited. Experiment has demonstrated, time and again, the remarkable resistance of the human body to extremes of heat, even under long exposure. It is a question, then, how far we may be justified in our efforts to reduce temperature that involve either prostration or unnecessary perturbation. I can recall several cases of this disease, making good recoveries, in which the average of minimum temperature did not fall below 105 degrees for eight days; and experience has certainly taught me to look with more anxiety to the pulse for a warning of peril than to the thermometer. Sponging, either cold, tepid, or warm, over the whole body, and continued, more or less persistently during much of the afternoon and evening, when the maximum range is being reached, has rarely failed, in my practice, to moderate the temperature and promote comfort and sleep. The end is thus attained without unnecessary fatigue, dangerous movement, or even mental shock. In addition, I have sometimes given one or two doses, five grains each, of antikamnia and quinine, during the afternoon and evening, for the purpose of anticipating and prolonging the remission, softening the skin and securing quiet sleep.

When the patient begins to show signs of muscular prostration, and the force of the heart's systole is apparently failing, my main reliance for years has been placed on strychnia given in small doses and often. I feel sure that many a flagging heart may be sustained by this remedy through the trying ordeal of the last weeks of an attack. On the subject of alcohol, I have nothing to add to what you already know, except to say that in late years my doses of whisky are much smaller than they used to be, and that I expect less from it now than formerly.

Of the manifold incidents and accidents presenting themselves in the course of the disease, I have not the time left to speak. A consideration of these would make up really the most interesting and important part of a paper on this subject. It is exactly here that the best judgment of the physician is a most severely taxed and his skill exemplified. It may be said, in a general way, that the doctor who is the most watchful, the quickest to apprehend the peril of complications and the most resourceful in wisely meeting emergencies, will have the best success. But the field is too broad to be entered. I want to call attention, however, to one not very frequent but occasional distressing symptom in the nervous subjects suffering with this disease—more common now than formerly—insomnia. Patients do not always sleep who appear to sleep; are not always properly rested by the apparent sleep they do get, because the sleep is not sufficiently profound. I shall never forget the luxury that a few doses of opium brought to me in the latter stages of this fever. I will add a passing reference to that common and serious event which so often complicates mild as well as severe cases—hæmorrhage from the bowel—and say I have found large doses of opium the most useful treatment. The exigency is often urgent and pressing; there is no time to waste on astringents. Peristalsis must be profoundly controlled, and the heart's action also. These threatening cases often do well, eventually, but it happens the only case of typhoid fever I have lost during the past four years was a very mild case that died speedily from this complication. I had meant to say something about relapses and recrudescences of fever, so common in typhoid, but this paper has already exceeded its legitimate limits. I can only repeat, it was not designed to be in any way complete or exhaustive; and I submit it merely as a text for this afternoon's discussion, that we may wisely compare views and experiences and, thereby, further fit ourselves to measure up to the serious responsibilities of our profession.

---

STRYCHNINE IN PREGNANCY.. Olenyn (*Protocol of the Medical Society of Tombow* for 1894) has successfully used strychnine in sixteen cases for the correction of weak labor-pains in doses of $\frac{1}{32}$ to $\frac{1}{25}$ grain twice daily, at intervals, during the last six or eight weeks of pregnancy. Four of these cases were anæmic primiparæ from 19 to 32 years of age with weak muscles; three were multiparæ under 30 years, with habitual weak labor-pains; four suffered from chronic metritis and had been pregnant at intervals of from three to twelve years; one patient had a small uterine fibroid; two had flabby uterus and relaxed abdominal walls; one had tertiary syphilis and general debility, and another diseased appendages with hysteria. In two primiparæ the forceps had to be used, and in one the child was dead; but in all the other cases delivery was rapid and regular and the children lived. The third stage lasted from ten to twenty minutes and *post-partum* contraction of the uterus was excellent.—*University Medical Magazine.*

